# Long-Term Suboptimal Anticoagulation in a Patient With Double Mechanical Heart Valves Leading to Stroke and Heart Failure

**DOI:** 10.7759/cureus.73611

**Published:** 2024-11-13

**Authors:** Gurvinder Sidhu, Pratyush Pradeep, Dyutima Singh, Bara Erhayiem

**Affiliations:** 1 Cardiology, Nottingham University Hospitals National Health Service (NHS) Trust, Nottingham, GBR; 2 Internal Medicine, Calderdale and Huddersfield National Health Service (NHS) Foundation Trust, Huddersfield, GBR

**Keywords:** heart failure with reduced ejection fraction, mechanical heart valves, oral anticoagulation, stroke, surgical case reports

## Abstract

Mechanical heart valve replacements are highly durable and effective but come with a substantial requirement for lifelong anticoagulation therapy to prevent thromboembolic complications. Unlike biological valve replacements, which typically require less rigorous anticoagulation, mechanical valves - particularly in the aortic and mitral positions - present a higher risk for clot formation, necessitating strict adherence to anticoagulation regimens. This case report examines a 59-year-old male with double mechanical heart valve replacements who experienced poor compliance with anticoagulation therapy for over 30 years, ultimately leading to significant health complications. Despite his long-standing non-compliance, the patient initially avoided severe thromboembolic events until he suffered a stroke in 2022, followed by the onset of severe heart failure due to persistent suboptimal anticoagulation. His case is unique, given the prolonged lack of adherence to anticoagulation therapy, and highlights the critical need for consistent anticoagulation management in patients with mechanical heart valves. We explore the challenges of managing anticoagulation in complex cases and underscore the importance of consistent adherence to anticoagulation therapy, multidisciplinary intervention, and patient education in improving patient outcomes.

## Introduction

Anticoagulation therapy is crucial for patients with mechanical heart valve replacements to prevent thromboembolic events and other complications, including valve thrombosis and an increased susceptibility to developing infective endocarditis [1]. The mainstay of treatment for these patients is indefinite anticoagulation with a vitamin K antagonist (VKA) [2]. A 1994 meta-analysis showed that VKA therapy significantly reduced the rate of major systemic embolization (from 4.0 to 1.0 events per 100 patient-years), total thromboembolism risk (from 8.6 to 1.8 events per 100 patient-years), and risk of valve thrombosis (from 1.8 to 0.2 events per 100 patient-years) compared to no antithrombotic therapy [2].

Mechanical heart valve replacement has evolved significantly since the 1950s when the concept of mechanical heart valve replacement started to take shape. Initial attempts involved rudimentary ball-and-cage designs [1]. However, the real breakthrough came in the 1960s when American surgeon Albert Starr and engineer Lowell Edwards collaborated to develop the first successful mechanical heart valve. The 1990s ushered in further advancements with the introduction of bi-leaflet mechanical heart valves, exemplified by the St. Jude Medical valve.

Our case report details a 59-year-old male with double mechanical heart valve replacements who experienced significant issues with anticoagulation therapy compliance over three decades. Despite his non-compliance, he avoided major thromboembolic complications until suffering a stroke in 2022 and later presenting with severe heart failure (HF). This case underscores the critical need for consistent anticoagulation management and highlights the potential consequences of suboptimal adherence to clinical guidelines.

## Case presentation

**Figure 1:**
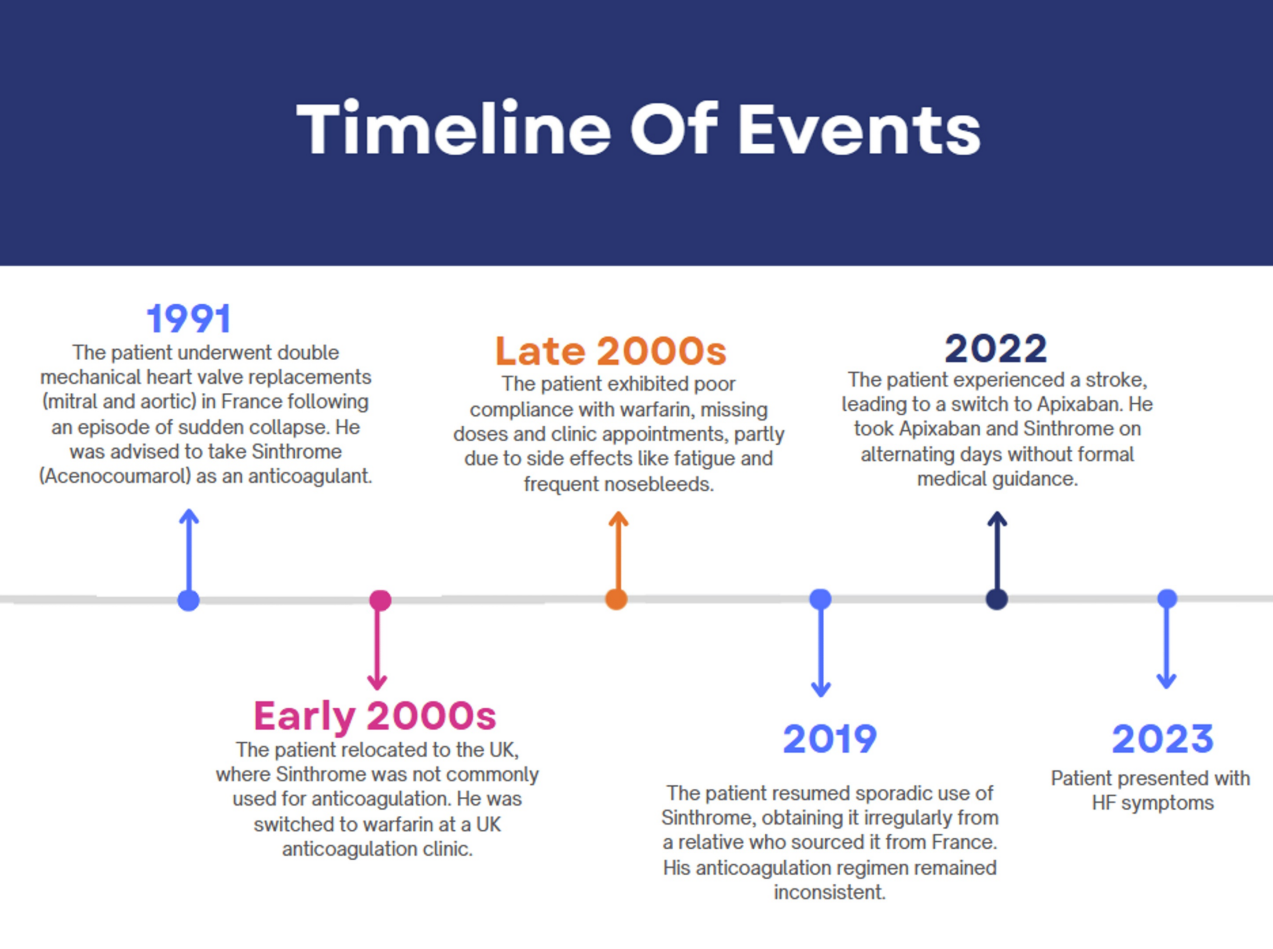
Timeline of events for this case presentation spanning several decades. HF, heart failure

Our patient, a 59-year-old male, underwent two mechanical heart valve replacements (mitral and aortic) in 1991 in France after an episode of sudden collapse. This is illustrated in Figure 1, our timeline of events. Following these procedures, his surgeon recommended Sinthrome (Acenocoumarol) as an anticoagulant, suggesting that it could ensure valve longevity for up to 50 years. Despite this advice and having no side effects, our patient would still inconsistently take their medicine by their own admission due to simply forgetting to do so. Upon relocating to the UK in the early 2000s, he encountered an additional obstacle - Sinthrome was not widely used for anticoagulation in this country in spite of being licensed.

In the absence of Sinthrome, he transitioned to warfarin therapy under the care of an anticoagulation clinic, only intermittently maintaining it for five years, which was evidenced by clinic records citing “poor compliance” and missed appointments. Unfortunately, the warfarin regimen took a toll on his quality of life, causing fatigue and frequent nosebleeds. He distinctly recalled being cautioned about the life-threatening risks of valve thrombosis and stroke if he discontinued the medication. Struggling with financial pressure and unable to work effectively, he felt compelled to discontinue warfarin on his own. Regrettably, he did not recall being offered an alternative solution at the time, and indeed, there was no record of this at his clinic appointments. 

In 2019, he resumed sporadic Sinthrome use, relying on the limited supply provided by a first-degree relative, who would buy this for him from France. However, the intermittent nature of this therapy made it challenging to maintain consistent anticoagulation. The turning point came in 2022 when he experienced a stroke. This led to the initiation of single Apixaban therapy, which was an oversight. Apixaban posed a new set of challenges due to its incompatibility with mechanical heart valves. Despite this, our patient chose to take Sinthrome for one day, followed by one day of Apixaban, without any formal medical advice to do so.

In the later months of 2023, he presented to his GP with symptoms of paroxysmal nocturnal dyspnea (PND), orthopnea, increasing shortness of breath (SOB) on exertion, and bilateral leg swelling. An outpatient echocardiogram was arranged, which revealed severe left ventricular systolic dysfunction (LVSD) with an estimated ejection fraction (EF) of approximately 10%. It was after this appearance that our patient was admitted to the cardiology ward for further treatment of his new HF diagnosis, and it was discovered that he had been on suboptimal anticoagulation therapy for decades.

Investigations

On admission, echocardiography revealed severe left ventricular systolic dysfunction with an EF of 10% and dilated ventricles, though the prosthetic valves were functioning normally. Chest X-ray (CXR) showed pulmonary edema.

The electrocardiogram (ECG) indicated left ventricular hypertrophy alongside rate-controlled atrial fibrillation. Blood results, detailed in Table 1, highlighted an elevated B-type natriuretic peptide (BNP) of 14,624, a creatinine level of 127, and an initial international normalized ratio (INR) of 1.2, which had increased to 5.3 by discharge. Valve fluoroscopy displayed a "fluttering" motion of the anterior leaflet of the mitral valve replacement, yet there were no functional concerns observed with either the mitral or aortic prosthetic valves.

**Table 1:** BNP, INR, and creatinine results at admission and discharge BNP, B-type natriuretic peptide; INR, international normalized ratio

Test	Result	Normal (target) range	Units
BNP	14,624	<100	pg/mL
Creatinine	127	59-104	µmol/L
INR on admission	1.2	0.8-1.1 (2.5-3.5)	Ratio
INR on discharge	5.3	0.8-1.1 (2.5-3.5)	Ratio

Treatment

The patient’s treatment plan involved a tailored, guideline-based medication regimen for HF with reduced ejection fraction, supplemented by loop diuretics for symptom relief. Valve fluoroscopy was performed to assess his prosthetic valves for potential thrombus formation. For anticoagulation, he initially received twice-daily enoxaparin injections and was later discharged with a structured warfarin regimen, including a loading dose of 9 mg in line with local guidelines and supported by regular follow-up with the anticoagulation clinic. Before discharge, the HF nursing team provided detailed education on managing and titrating his HF medications, ensuring he was prepared for at-home care. Additionally, a multidisciplinary plan was established: if warfarin proved insufficient, a collaborative team meeting would explore alternative anticoagulation strategies to address his specific needs comprehensively.

Outcomes and results 

Our patient was readmitted on February 2, 2024, following a recurrence of HF symptoms compounded by non-compliance with cardiac medications. Clinical assessment, including a CXR, revealed signs indicative of cardiac overload.

Notably, our patient's INR was measured at 1.82, indicating continued suboptimal compliance with warfarin therapy. During his short stay, he was diuresed; his Furosemide was switched to Spironolactone, and he was subsequently discharged also after restarting warfarin.

Our patient failed to attend a follow-up in April, which highlighted ongoing challenges in managing his care.

## Discussion

The case we present here provides a stark contrast to the conventional guidelines and practices related to anticoagulation therapy in patients with mechanical prosthetic heart valves. It is worthwhile to draw similarities between this case and another in which a 46-year-old man with a single mechanical aortic valve faced no complications after 33 years without anticoagulation therapy per se, only taking Aspirin and Digoxin [3]. In this, the patient's mechanical aortic valve had been functioning well for over three decades with Aspirin alone, contrary to established guidelines mandating anticoagulation therapy. 

**Figure 2:**
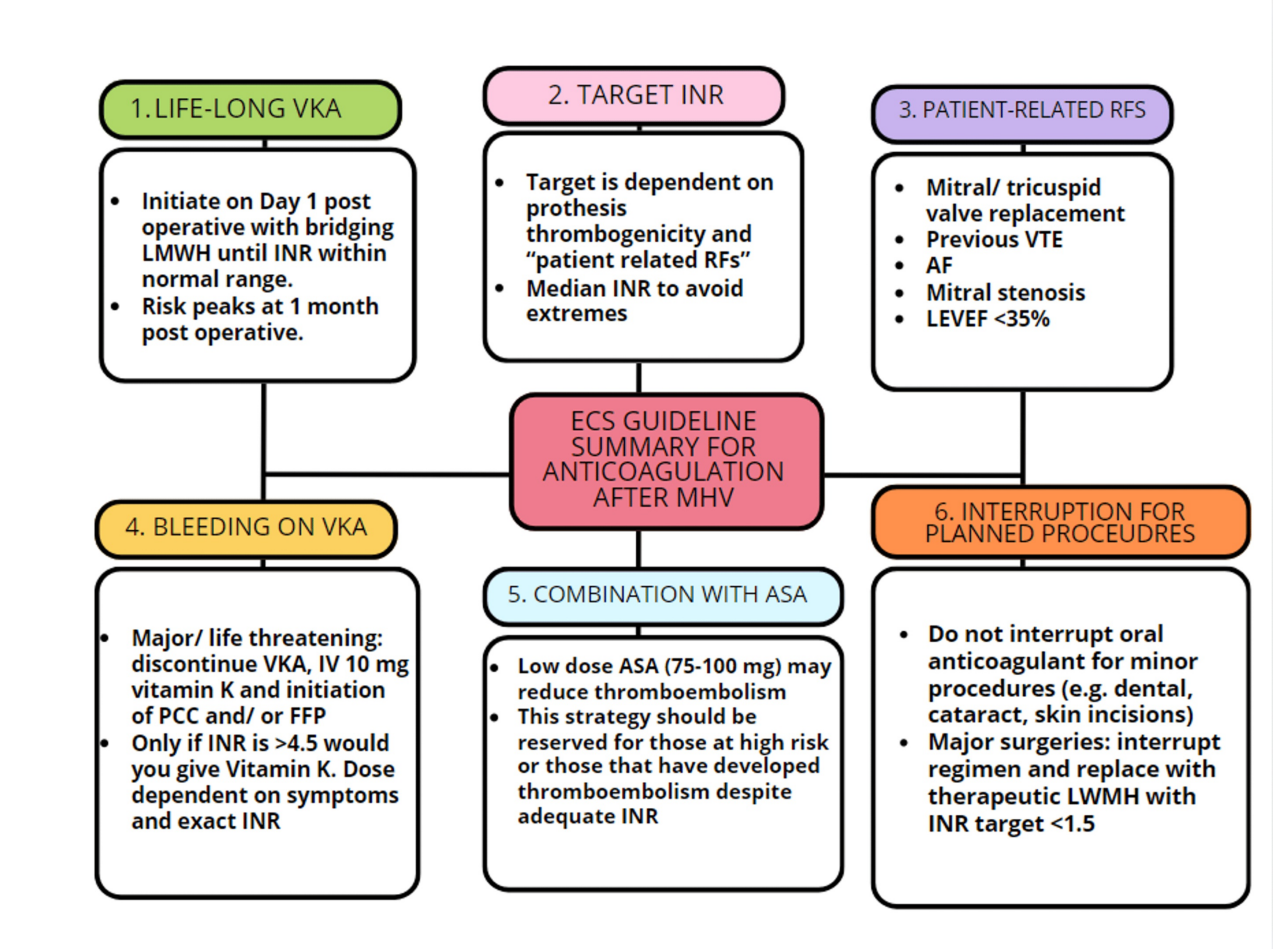
Summary of European Society of Cardiology (ESC) 2021 management guidelines for anticoagulation in mechanical valves. AF, atrial fibrillation; ASA, acetylsalicylic acid (aspirin); FFP, fresh frozen plasma; INR, initial international normalized ratio; LMWH, low-molecular-weight heparin; LVEF, left ventricular ejection fraction; PCC, prothrombin complex concentrate; RFs, risk factors; VKA, vitamin K antagonist; VTE, venous thromboembolism

Existing European Society of Cardiology (ESC) 2021 management guidelines for anticoagulation in mechanical valves emphasize the lifelong necessity of treatment with VKA therapy (Figure 2) guided by the INR for patients with mechanical prosthetic heart valves [4]. They recommend initiating VKA therapy on the first postoperative day, coupled with bridging therapy, typically involving either unfractionated heparin (UFH) or low-molecular-weight heparin (LMWH) until a therapeutic INR is achieved [4]. The target INR for patients with mechanical valves is recommended to be based on prosthesis thrombogenicity and patient-related risk factors. While these guidelines propose a specific target range for each category, it is essential to aim for a median INR value rather than a range to ensure stability in anticoagulation therapy. High INR variability is linked to adverse events after valve replacement. 

Another area of interest in the case is that, as per the ESC guidelines for valvular disease, there is no specific preference for warfarin over Acenocoumarol, leaving room for the use of Acenocoumarol where appropriate. Cost considerations are also relevant, with Acenocoumarol being approximately 6.5 times more expensive than warfarin, which can impact the overall treatment cost. On reflection, perhaps this was an opportunity that was missed in providing an alternative, licensed method of anticoagulation for our patient. In addition, the absence of specific criteria in existing guidelines regarding the choice between these anticoagulants highlights the need for ongoing research and guideline updates.

## Conclusions

In conclusion, we can reflect on the complexities highlighted by the case of our patient. Despite having two mechanical heart valve replacements, our patient experienced a significant gap in optimal anticoagulation between 1991 and 2022. This lapse in appropriate anticoagulation management led to a stroke in 2022 (and subsequent initiation of inappropriate Apixaban therapy) and a possible new presentation of HF secondary to valve thrombosis. Although the latter was not confirmed, this underscores the importance of consistent adherence to anticoagulation guidelines, especially in patients with mechanical prosthetic valves. Exceptional cases like that of our patient and the aforementioned case of the 46-year-old challenge our understanding that patients may remain clinically well for long periods of time. However, it is essential to balance such observations with the well-established benefits of guideline-recommended anticoagulation in preventing thromboembolic complications.

For patients with mechanical heart valves, consistent adherence to anticoagulation therapy is essential to prevent thromboembolic events, which can have serious or even life-threatening consequences. Comprehensive patient education plays a critical role in supporting compliance, as understanding the risks of non-adherence empowers patients to follow their regimens more diligently. Regular follow-up and support from anticoagulation clinics further improve patient outcomes, and in cases where non-adherence occurs, exploring the underlying causes is key to addressing any barriers to adherence. Effective management also relies on a coordinated, multidisciplinary approach, with clear communication between healthcare providers and the patient to manage complex cases optimally. In our patient’s case, additional discussions between specialists might have highlighted licensed alternative anticoagulation options, such as Sinthrome, offering a tailored approach to meet his unique needs.
